# Cognitive deficits and anxiety induced by diisononyl phthalate in mice and the neuroprotective effects of melatonin

**DOI:** 10.1038/srep14676

**Published:** 2015-10-01

**Authors:** Ping Ma, Xudong Liu, Jiliang Wu, Biao Yan, Yuchao Zhang, Yu Lu, Yang Wu, Chao Liu, Junhui Guo, Eewa Nanberg, Carl-Gustaf Bornehag, Xu Yang

**Affiliations:** 1 Hubei Province Key Laboratory on Cardiovascular, Cerebrovascular and Metabolic Disorders, Hubei University of Science and Technology, Xianning 437100, China; 2 Lab. of Environmental Biomedicine, Hubei Key Laboratory of Genetic Regulation and Integrative Biology, College of Life Science, Central China Normal University, Wuhan 430079, China; 3 Department of Health Sciences, Karlstad University, Karlstad, Sweden; 4 Department of Food science and Engineering, Moutai University, Renhuai 564500, China

## Abstract

Diisononyl phthalate (DINP) is a plasticizer that is frequently used as a substitute for other plasticizers whose use is prohibited in certain products. *In vivo* studies on the neurotoxicity of DINP are however, limited. This work aims to investigate whether DINP causes neurobehavioral changes in mice and to provide useful advice on preventing the occurrence of these adverse effects. Behavioral analysis showed that oral administration of 20 or 200 mg/kg/day DINP led to mouse cognitive deficits and anxiety. Brain histopathological observations, immunohistochemistry assays (cysteine-aspartic acid protease 3 [caspase-3], glial fibrillary acidic protein [GFAP]), oxidative stress assessments (reactive oxygen species [ROS], glutathione [GSH], superoxide dismutase [SOD] activities, 8-hydroxy-2-deoxyguanosine [8-OH-dG] and DNA-protein crosslinks [DPC]), and assessment of inflammation (tumor necrosis factor alpha [TNF-а] and interleukin-1 beta [IL-1β]) of mouse brains showed that there were histopathological alterations in the brain and increased levels of oxidative stress, and inflammation for these same groups. However, some of these effects were blocked by administration of melatonin (50 mg/kg/day). Down-regulation of oxidative stress was proposed to explain the neuroprotective effects of melatonin. The data suggests that DINP could cause cognitive deficits and anxiety in mice, and that melatonin could be used to avoid these adverse effects.

Diesters of 1, 2-benzenedicarboxylic acid (phthalic acid), commonly referred to as phthalates, are man-made chemicals widely used by the chemical industry in the manufacture of polymers^1^. This group of chemicals has a wide spectrum of industrial applications and these chemicals appear, ultimately, in a wide range of consumer products, such as automotive parts, building materials, cosmetics, and toys, as well as being used in food processing and medical applications^2^.

Because phthalates are not chemically bound to the polymers, there is concern that they can leach out from the polymer matrix during use^3^. Some studies have shown that Di (2-ethylhexyl) phthalate (DEHP), di-n-butyl phthalate (DBP), and benzylbutyl phthalate (BzBP) disrupt reproductive tract development in male rodents in an anti-androgenic manner to reduce fetal testicular testosterone production^4^. A number of non-reproductive conditions have also been reported in the literature, such as hepatic and renal effects, hepatocellular carcinoma, anovulation, and decreased fetal growth^5^. Because of the adverse effects of these phthalates, North America and Europe have promulgated some stringent government restrictions on the use of DEHP, DBP and BzBP in consumer products. These regulations have resulted in the replacement of DEHP, DBP and BzBP with other less toxic phthalates, especially diisononyl phthalate (DINP)^6^.

Similar to other phthalates, DINP is also not covalently bound to plastics. Thus, humans may be exposed to DINP via oral, dermal, and inhalation routes. Occupational exposure occurs primarily through inhalation and dermal contact, while consumer exposure is primarily via oral and dermal routes. More importantly, children may be exposed to higher levels of DINP than adults because infants and small children play with mouth toys and other articles that can contain DINP^7^. According to studies by some agencies, such as Health Canada and RIVM (The National Institute of Public Health and Environment), the estimated human daily intake is 70.7–204 μg/kg/day to 320 μg/kg/day^8^. Although DINP is regarded as a less toxic phthalate, the adverse health effects, including reproductive toxicity, organ toxicity, and carcinogenicity have been observed in previous studies. The European Union Risk Assessment Report (UC-JRC, 2003), and the European Chemicals Agency Report (ECHA, 2010) show that 200 mg/kg/day is a toxic dose of DINP^9^.

Mature neurons in the human nervous system cannot proliferate for self-repair, making this system particularly vulnerable to environmental pollutants. Although there are some studies into the toxic effects resulting from exposure to DINP, neurotoxicity data are limited. In this study, neurotoxicity of DINP was detected. After mice were orally exposed to DINP (0 mg/kg/day, 0.2 mg/kg/day, 2 mg/kg/day, 20 mg/kg/day, 200 mg/kg/day), we looked for behavioral changes using the Morris water maze [MWM] test and the Open field test [OFT], and tested for brain tissue damage using histopathological observations and immunohistochemistry assays. The key upstream events (reactive oxygen species [ROS], glutathione [GSH], superoxide dismutase [SOD] activity, DNA-protein crosslinks [DPC], 8-hydroxy-2-deoxyguanosine [8-OH-dG], tumor necrosis factor alpha [TNF-а] and interleukin-1 beta [IL-1β]) for the resulting damage were examined to explore possible mechanisms. Additionally, the neuroprotective effects of melatonin (Mel) were examined after DINP exposure. The primary goal of this work was to define any damage in the mouse brain after exposure to DINP, and to investigate whether melatonin could be used as an agent to protect against high doses of DINP (Fig. 1).

## Results

### DINP treatment resulted in cognitive deficits

**Figure 2:**
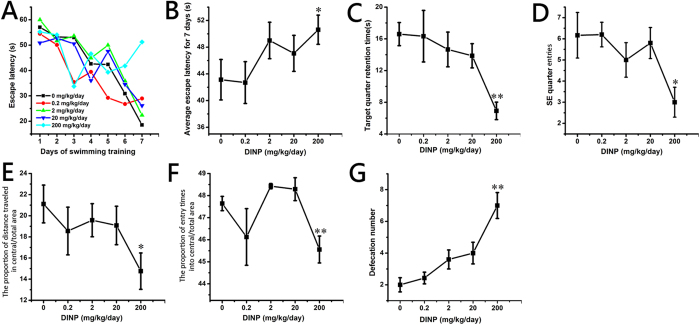
DINP treatment resulted in cognitive deficits and anxiety. (**A**) The escape latency for the 7 days training. (**B**) The average escape latency for 7 days training. (**C**) The swimming time in the target quadrant on the 14th day. (**D**) The number of target quadrant entries on the 14th day. (**E**) The proportion of distance traveled in central/total area. (**F**) The proportion of entry times into central/total area. (**G**) The defecation number. Repeated-measures ANOVA followed by a Tukey test was used in the analysis of MWM escape latencies. A one-way ANOVA followed by a Tukey test was used to analyze all other data. *P < 0.05; **P < 0.01, compared with the control group (0 mg/kg/day DINP). In MWM, the number of mice in 0.2, 2 and 200 mg/kg/day group was 6, other groups was 7. In OFT, the number of mice in all groups was 7.

In neuroscience, MWM is a behavioral test that is designed to evaluate the cognitive abilities of animals. As shown in Fig. 2, after 7 days (from the 6th to 12th day) of training to find the escape platform, the escape latency (the time it takes to find the escape platform) was reduced in each group (Fig. 2A). The escape latency for the control group mice showed the greatest decrease over the 7 days period, whereas that for the 200 mg/kg/day group showed the least decrease. That is to say, the mice in the control group learned the fastest while the mice in the 200 mg/kg/day group learned the slowest. A significant increase (*P *< 0.05) in the average escape latency for 7 days was detected in the mice from the 200 mg/kg/day group, compared with the mice from the control group (Fig. 2B). The mice received no training on the 13th day (the forgetting-period), and then the spatial memory ability of the mice after DINP exposure was evaluated on the 14th day. On the 14th day, the time that each group spent swimming in the target (SE) quadrant was compared to the time spent by the control group (Fig. 2C). The time spent by the mice in the 200 mg/kg/day group was significantly decreased (*P *< 0.01), and the frequency of entry (times of one test) into the target quarter showed the same trend (Fig. 2D, *P *< 0.05). From the swimming pathway data from the 14th day (Figure S1A), it is clear that the mice in the control groups swam purposefully, in an orderly manner, and were focused on the target SE quadrant, whereas the pathways of the mice in the 200 mg/kg/day group were irregular and showed little purpose.

### DINP treatment resulted in anxiety

The OFT is one of the most widely used measures of rodent behavior, and is frequently used to assess the anxiety-like emotions of animals by evaluating their movement in an open field. As shown in Fig. 2E,F, we observed significant decreases in central/total area distance (*P *< 0.05) and central/total area entries (*P *< 0.01) in the 200 mg/kg/day group when compared with mice from the control group. In addition, the movement pathways of mice in the 200 mg/kg/day group were concentrated in the border area (Figure S1B). The defecation number (Fig. 2G) also showed significant increases (*P *< 0.01) in the 20 and 200 mg/kg/day groups.

### Histopathological changes were observed in brains after DINP treatment

**Figure 3:**
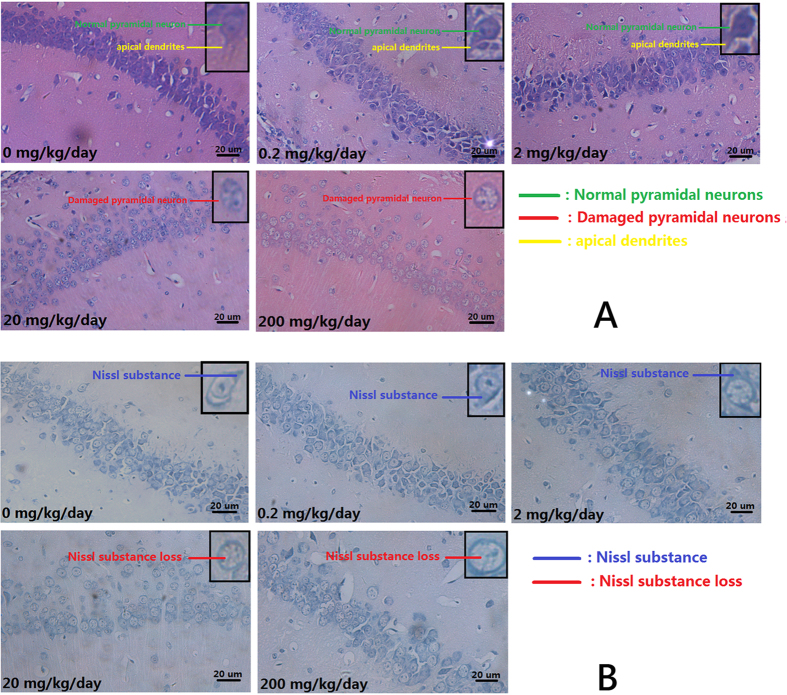
Histopathological changes were observed in brains after DINP treatment. (**A**) H&E staining. Normal pyramidal neuron (green line); Damaged pyramidal neuron (red line); Apical dendrites (yellow line). (**B**) Nissl staining. Nissl substance (blue line); Nissl substance loss (red line).

Once the MWM test and the OFT were completed, the brains of the mice were collected for histological assessment. From the H&E stains (Fig. 3A), we observed that the pyramidal cells in the hippocampus CA_1_ region of the control group mice were neatly arranged, sharp edged, polygonal-shaped cells. Pathological changes were observed in the brains of mice exposed to to increasing concentrations of DIDP, ranging from loose and disordered arrangements of cells through swelling deformations of cells to the shortening or even to the disappearance of apical dendrites. Nissl staining showed (Fig. 3B) increasing exposure concentrations caused loss of Nissl substance in cells when compared with the cells from the control group, which had plenty of Nissl substance.

### Expression of caspase-3 and GFAP in brains were up-reguated after DINP treatment

**Figure 4:**
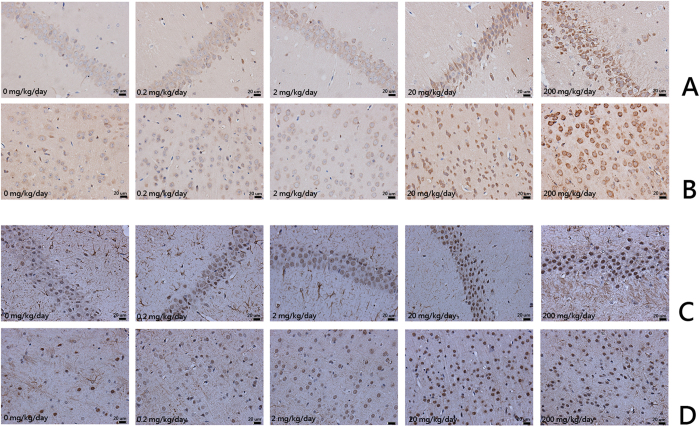
Expression of caspase-3 and GFAP in brains were up-reguated after DINP treatment. The coronal sections were cut at Bregma-3.8 mm. Representative images of the expression of (**A**) Caspase-3 in the CA_1_ region; (**B**) Caspase-3 in the cerebral cortex; (**C**) GFAP in the CA_1_ region; (**D**) GFAP in the cerebral cortex. Analyses of caspase-3 and GFAP expression levels according to average optical density.

Immunohistochemical analyses were conducted to detect the expression of caspase-3 and GFAP in brain tissue. Caspase-3 and GFAP were both expressed in cells of the hippocampus CA_1_ region and the cerebral cortex. Expression of these proteins was up-regulated in the DINP-treated group compared with the control groups (Fig. 4). The average optical density was also measured. We noted that the DINP-treated groups presented an increase in the content of caspase-3 and GFAP. Compared with the control group, the 20 or 200 mg/kg/day DINP exposure groups showed significantly enhanced levels of caspase-3 and GFAP both in the CA_1_ region of the hippocampus and in the cerebral cortex (Table 1).

### DINP treatment increased the oxidative stress level in brains

**Figure 5:**
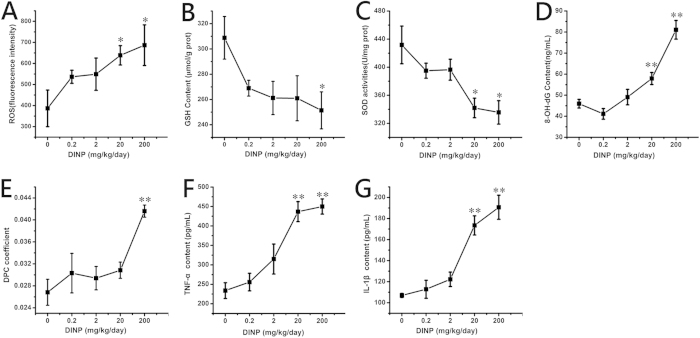
DINP treatment increased the oxidative stress level and induced inflammation in brains (n = 7). (**A**) The relative fluorescence of ROS. (**B**) The concentration of GSH. (**C**) The activity of SOD. (**D**) The level of 8-OH-dG in the brain. (**E**) The DPC coefficient in the brain. (**F**) The content of TNF-а. (**G**) The content of IL-1β. Data were analyzed by a one-way ANOVA followed by a Tukey test. *P < 0.05; **P < 0.01, compared with the control group (0 mg/kg/day DINP).

After DINP exposure, significant increases (*P *< 0.05) in ROS levels were observed in the 20 and 200 mg/kg/day groups (Fig. 5A). GSH content of the 200 mg/kg/day group decreased significantly (*P *< 0.05) compared with that of the control group (Fig. 5B), and SOD activity levels showed a similar trend, i.e., levels decreased significantly (Fig. 5C, *P *< 0.05) in the 20 and 200 mg/kg/day groups. DPC and 8-OH-dG were used to measure the DNA damage associated with oxidative stress. As shown in Fig. 5D,E, both 8-OH-dG (20 and 200 mg/kg/day groups) and DPC (200 mg/kg/day group) increased significantly (*P *< 0.01) after DINP exposure.

### DINP treatment induced inflammation in brains

Figure 5F,G, show that the expression of TNF-α and IL-1β in the 20 and 200 mg/kg/day groups increased significantly (*P *< 0.01).

### The protective effects of melatonin (Mel)

**Figure 6:**
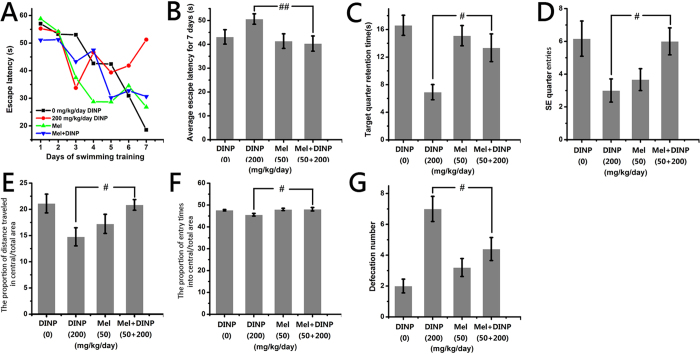
Mel’s protective effects on the cognitive deficits and anxiety induced by DINP exposure. (**A**) The escape latency for the 7 days training. (**B**) The average escape latency for 7 days training. (**C**) The swimming time in the target quadrant on the 14th day. (**D**) The number of target quadrant entries on the 14th day. (**E**) The proportion of distance traveled in central/total area. (**F**) The proportion of entry times into central/total area. (**G**) The defecation number. Repeated-measures ANOVA followed by a Tukey test was used in the analysis of MWM escape latencies. A one-way ANOVA followed by a Tukey test was used to analyze all other data. ^#^*P *< 0.05; ^##^*P *< 0.01, compared with the 200 mg/kg/day DINP. In MWM, the number of mice in 200 mg/kg/day group was 6, other groups was 7. In OFT, the number of mice in all groups was 7.

The mice that had Mel administered in addition to DINP were observed to spend less time (*P *< 0.01) finding the escape platform (Fig. 6A,B) in the MWM test, they found the target quadrant more easily, spent longer time (*P *< 0.05) there (Fig. 6C,D), and swam with more purpose (Figure S1A) when compared with the 200 mg/kg/day DINP only group. In the OFT, the DINP+Mel group mice entered the central area more often (Fig. 6E,F, *P *< 0.05 and S1B), and the defecation number was significantly reduced (Fig. 6G, *P *< 0.05) as compared to the 200 mg/kg/day DINP only group.

**Figure 7:**
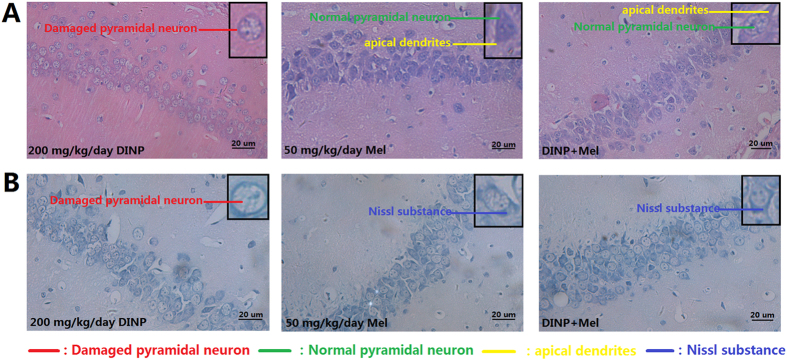
Mel’s protective effects on brain tissue damage induced by DINP exposure. (**A**) H&E staining. Normal pyramidal neuron (green line); Damaged pyramidal neuron (red line); Apical dendrites (yellow line). (**B**) Nissl staining. Nissl substance (blue line).

**Figure 8:**
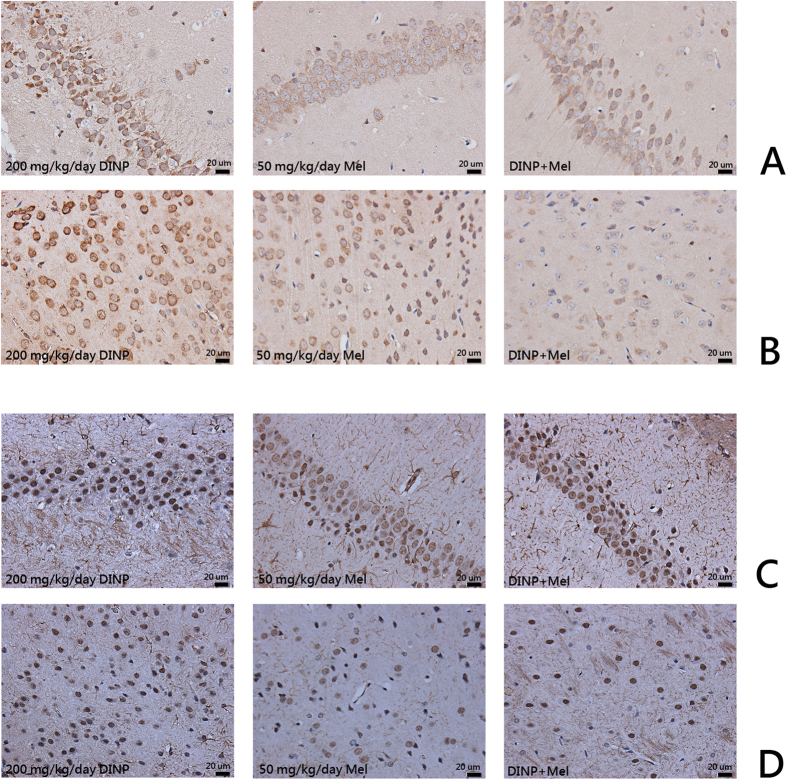
The inhibitory effects of Mel on caspase-3 and GFAP up-regulated induced by DINP exposure. The coronal sections were cut at Bregma-3.8 mm. Representative images of the expression of (**A**) Caspase-3 in the CA_1_ region; (**B**) Caspase-3 in the cerebral cortex; (**C**) GFAP in the CA_1_ region; (**D**) GFAP in the cerebral cortex. Analyses of caspase-3 and GFAP expression levels according to average optical density.

Histological observations and an immunohistochemical assay after Mel administration showed that although the DINP+Mel group had sustained cell damage,,this damage was much less than that seen in the 200 mg/kg/day DINP alone group. The majority of cells in DINP+Mel group remained intact and therefore stained homogeneously (Fig. 7A). Nissl staining results (Fig. 7B) showed that nissl substance loss was lower in the mice from the DINP+Mel group. When mice were treated with Mel, the expression of caspase-3 and GFAP in brain tissues was reduced (Fig. 8, Table 2).

**Figure 9:**
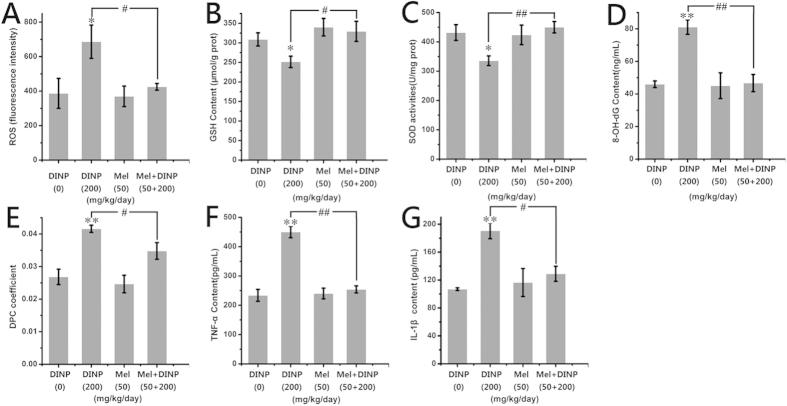
The inhibitory effects of Mel on oxidative stress induced by DINP exposure (n = 7). (**A**) The relative fluorescence of ROS. (**B**) The concentration of GSH. (**C**) The activity of SOD. (**D**) The level of 8-OH-dG in the brain. (**E**) The DPC coefficient in the brain. (**F**) The content of TNF-а. (**G**) The content of IL-1β. Data were analyzed by a one-way ANOVA followed by a Tukey test. *P < 0.05; **P < 0.01, compared with the control group (0 mg/kg/day DINP), ^#^*P *< 0.05; ^##^*P *< 0.01, compared with the 200 mg/kg/day DINP.

The amount of ROS generated in the DINP+Mel group was significantly less (*P *< 0.05) than that in the 200 mg/kg/day DINP alone group as can be seen in Fig. 9A. The GSH levels in the DINP+Mel group were significantly higher (*P *< 0.05) than that of the 200 mg/kg/day DINP alone group (Fig. 9B). The activities of SOD showed a similar trend to GSH, with the activities of the DINP+Mel group increasing significantly (Fig. 9C, *P *< 0.01). The damage to DNA was also mitigated by 8-OH-dG and DPC in the DINP+Mel group, which decreased significantly compared with that of the 200 mg/kg/day DINP alone group (Fig. 9D, *P *< 0.01 and 9E, *P *< 0.05). The release of TNF-α and IL-1β in the DINP+Mel group also decreased (Fig. 9F,G) compared with the 200 mg/kg/day DINP alone group.

## Discussion

DINP has been widely used as a substitute for DEHP by the chemical industry in the manufacture of polymers and a variety of consumer products. Although DINP is a less toxic phthalate, it has been shown to exhibit antiandrogenic activity as well as liver and kidney toxicity. This work aimed to understand DINP exposure-induced toxicology in the brain, and to explore a strategy to protect people from these adverse effects.

In our work, MWM, and OFT were used to evaluate the cognitive deficits and anxieties induced by DINP exposure. The MWM required the mice to find a hidden platform just below the surface of a pool and to remember its location according to prior spatial memory training^10^,^11^. We observed that mice from the 200 mg/kg/day DINP group took longer to find the submerged escape platform: the number of entries into, and time spent in the target SE quadrant decreased; and their swimming pathways became irregular and without purpose. From this we infer that the cognitive abilities of mice after exposure to DINP were reduced. The OFT is widely used and is an important approach for the assessment of anxiety-like behavior, exhibited by the movement of mice in the open field. In this test the distance and paths that mice travel are recorded. Generally, OFT mice with anxiety-like behavior will focus on the border area, and an increase in the number of times they defecate has been reported^12^,^13^,^14^. In the OFT, a decrease in central distances covered and the number of central area entries made indicate that the pathways were concentrated in the border areas, suggesting that DINP exposure (200 mg/kg/day) could cause a reduction in the locomotor activity of mice and could induce anxiety. Anxiety-like behaviour can also be inferred from the increase in defecation number.

There are many studies showing that the hippocampus plays a critical role in memory. The hippocampus comprises distinct regions, sometimes referred to as CA_1_–CA_4_^15^. The literature suggests that there is a good correlation between the function of the pyramidal cells of region CA_1_ and memory^16^. More recent studies have focussed on the hippocampus as being responsible for mood disorders, and have shown that anxiety may result from structural changes in this region^17^.

Histological observations of mouse brains showed damage to the pyramidal cells in the CA_1_ region after DINP exposure at certain concentrations. This damage was characterized by loose and disordered arrangements of cells, swelling deformations in cell shape, shortening or even disappearance of apical dendrites and Nissl substance loss. The reduced cognitive abilities and increase in anxiety demonstrated by mice after DINP exposure may be the result of this cell damage.

The release of apoptosis factors and mitochondrial perturbation can induce apoptosis. For example, caspases have proteolytic effect and cleave proteins at aspartic acid residues. Activated caspases have an irreversible commitment towards cell death. Therefore, in apoptosis mechanisms caspase-3 plays a pivotal role^18^. According to the results of the immunohistochemical assay, the expression of caspase-3 increased in both the cells of the CA_1_ region and in the cerebral cortex. These results indicated that after DINP exposure the occurrence of apoptosis was detected in mice brains, and that exposure to DINP induces brain tissue damage.

Accumulating evidence suggests that astrocytes are associated with the pathology and development of neuro-degenerative diseases. Astrocyte activation is the common pathology characteristic. Astrocytes contribute to a variety of functions of neurons, including supporting and protecting motor neurons. But when astrocytes are activated, the shape and function of the motor neuron is changed. Crosstalk between astrocytes and the motor neuron becomes disordered, and this can accelerate the death of the motor neuron^19^. GFAP is a unique composition of intermediate filaments in mature astrocytes, and is widely used as a marker for astrocytes. When astrocytes are activated, the expression of GFAP increases sharply^20^,^21^. In our study, we observed that the expression of GFAP both in the cells of CA_1_ region and in cerebral cortex was increased. After DINP exposure, astrocytes were activated, and this could also partly account for the behavioral changes shown by mice after DINP exposure.

Oxidative stress is defined as a disturbance in the balance between the production and elimination of ROS^22^. ROS Elimination is related to tissue damage, and is closely associated with further damage to all cell components including DNA, proteins, and lipids^23^. The central nervous system has a high oxygen consumption and low antioxidant defense activity^24^ because of its special structure, and it is therefore reasonable for us to examine whether oxidative stress is a cause of mouse brain damage. Hence, related biomarkers and associated damage in mouse brains were measured to determine whether oxidative stress was involved in brain damage. In our study we analyzed ROS, GSH, SOD, 8-OH-dG and DPC to explore the mechanisms underlying the damage to mice brains that is induced by DINP exposure. ROS are produced by cellular metabolism^25^ and are the most important biomarker for oxidative stress. ROS accumulation can lead to oxidative stress in tissues and induce further tissue damage. GSH is a ubiquitous tri-peptide that primarily functions by reacting with hydrogen peroxide, but it also scavenges other ROS molecules to prevent oxidation. GSH provides antioxidant protection in the aqueous phase of cellular systems^26^. SOD is an important enzyme, which can eliminate ROS molecules to protect cells^27^. Therefore, depletion of GSH and decreases in SOD activity are also usually regarded as measures of oxidative stress. High levels of ROS often lead to DNA damage. ROS-induced DNA damage includes single- or double-stranded DNA breaks, modifications to purine, pyrimidine, or deoxyribose, and to DNA crosslinks^28^. Guanines in DNA are particularly active residues, which are easily attacked by ROS to form 8-OH-dG. 8-OH-dG binds to thymidine rather than cytosine to cause transversion mutations^29^. Another measure of DNA damage, DPC, was also detected in this study. The covalent crosslinking of proteins to DNA presents a major challenge to DNA metabolic machinery^30^. Our results demonstrated significant increases in ROS as well as decreases in GSH and SOD activity, which collectively suggest that after DINP exposure (20 or 200 mg/kg/day), oxidative stress was induced in the mice brains. Significant increases in 8-OH-dG and DPC suggested possible genotoxicity that might be associated with oxidative stress after DINP exposure (20 or 200 mg/kg/day).

In a previous study in our laboratory^31^, we also found a link between oxidative stress and cognitive deficits and anxiety in mice, the difference from this study was that the oxidative stress in the central nervous tissue was induced by a single-walled carbon nanotubes. Although environmental pollutants for exposure were different, but oxidative stress downstream events were very similar, including the results of Morris water maze test, open-field test, immunohistochemical assay and brain histological assay, as well as the antioxidant neuroprotective effects.

The hierarchical oxidative stress hypothesis describes a system in which low levels of oxidative stress are associated with cytoprotective responses, e.g., those using antioxidant and detoxification enzymes, while high levels of oxidative stress can result in cell damage or even cell death^32^. High levels of oxidative stress can lead to pro-inflammatory effects such as induced expression of tumor necrosis factor alpha (TNF-α)^33^ and interleukin-1 beta (IL-1β)^34^. As shown in our results, the content of TNF-α and IL-1β in mouse brains increased significantly after exposure to high concentrations of DINP (20 and 200 mg/kg/day) suggesting that the inflammation seen in the mice brains was caused by DINP-induced oxidative stress.

Melatonin is a versatile molecule endowed with a broad range of physiological functions. The ROS quenching capability and blood-brain barrier permeability of melatonin suggest a possible role in neuroprotection^35^. Therefore, we used Mel as an inhibitor of oxidative stress caused by the DINP by testing a combination group (200 mg/kg/day DINP+50 mg/kg/day Mel)^36^, to demonstrate the neuroprotective effects of Mel at high-dose DINP exposure, and to elucidate possible protective mechanisms. These results demonstrated that administration of Mel significantly reduced damage; improved cognitive abilities; and reduced anxiety in the DINP-exposed mice. The levels of brain cell damage, expression of caspase-3 and GFAP, oxidative stress, and inflammation were also decreased. We can therefore conclude that Mel may protect against DINP induced brain damage by down-regulating the oxidative stress level.

**Figure 10:**
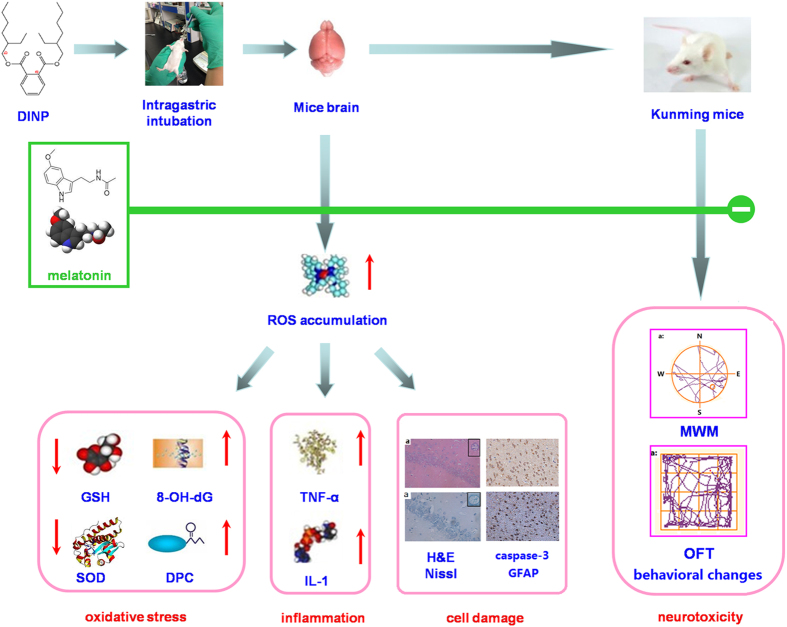
Potential mechanism of DINP-induced damage in mouse brains. DINP-induced adverse effects in mice brains occur through ROS generation and oxidative stress. Oxidative stress then induces cell damage in the brain, and learning memory and anxiety are altered are affected, whereas treatment with Mel protects cells by decreasing oxidative stress (Figure 10 was made by Xudong Liu and the different photographs of the mice were originally taken by Xudong Liu).

This study aimed to examine the effect of DINP on the brains of mice, and to explore whether Mel could be used as a protective agent. Our results suggest that high concentrations of DINP (20 or 200 mg/kg/day) decreased the cognitive abilities and induced anxiety in mice. We concluded that DINP resulted in oxidative stress that caused damage to the mouse brain. Furthermore, Mel was confirmed as a protective agent having been shown to decrease oxidative stress levels (Fig. 10).

## Methods

### Ethics statement

All experiments of this study were performed in accordance with relevant guidelines and regulations. The experimental procedures were approved by the Office of Scientific Research Management of the Central China Normal University, and the approval ID was CCNU-SKY-2011-008.

### Animals

Kunming mice (5–6 weeks old, 22 ± 2 g) were purchased from the Hubei Province Experimental Animal Center (Wuhan, China) and housed in standard environmental conditions (12-h light-dark cycle, 50–70% humidity, and 20–25 °C). Food and water were provided *ad libitum*. Seven mice were used in each group to minimize the number of experimental animals needed, while ensuring the validity of statistical analyses.

### Reagents and kits

DINP, 2′, 7′-dichlorodihydrofluorescein (DCFH-DA), 3-carboxy-4-nitrophenyl disulfide (DTNB), Hoechst 33258 and Mel were purchased from Sigma-Aldrich (St. Louis, MO, USA). All other chemicals were of the highest grade commercially available. The mouse superoxide dismutase (SOD) activity assay kit was purchased from Jiancheng (Jiancheng, Nanjing, Jiangsu, China). The mouse ELISA kit for 8-OHdG was purchased from R&D System (R&D System, Minneapolis, MN, USA). The mouse ELISA kits for TNF-α and IL-1β were purchased from eBioscience (eBioscience, San Diego, CA, USA). Rabbit anti-caspase-3-antibody, rabbit anti-GFAP- antibody, goat-anti-rabbit lgG-antibody, rabbit lgG peroxidase conjugated streptavidin-biotin complex (SABC-POD) kit and a diaminobenzidine (DAB) kit were obtained from Boster Bio-engineering (Boster Bio-engineering, Wuhan, China).

### Main equipment

The equipment and biomarkers used in our study are shown in Table 3.

### Experimental protocol and animal exposure

The aim of the study protocol was to simulate the human exposure route. All animals were exposed to DINP by intragastric intubation. Forty-nine mice were randomly assigned to one of seven groups: vehicle control (0.9% NaCl/Tween 80 1:1), 0.2, 2, 20, 200 mg/kg/day (DINP/Tween 80 1:1), 50 mg/kg/day Mel (the melatonin solution was 5 mg/mL), 200 mg/kg/day DINP+50 mg/kg/day Mel, in a dosing volume of 10 mL/kg body weight^35^. The Melatonin in our study was also administered orally (p.o.) as with the DINP. Melatonin was dissolved in 3% ethanol and diluted with sterile saline to a concentration of 50 mg/Kg/day (vortex and ultrasonic were used to promote dissolution). Different concentrations of DINP were prepared in Tween-80 (1:1 v/v) and diluted with sterile saline for the oral (p.o.) administrations. Animals received daily intragastric intubations via a metal gastric tube for 14 consecutive days (once daily between 7:00 and 8:30 AM), from the 6th to 12th day the animals performed the hidden-platform acquisition test (MWM), on the 13th day they were not subjected to any activity (the forgetting-period), and on the 14th day they were given the probe trial test and open-field test (OFT).

### MWM

The Morris water maze (MWM) is used to investigate the cognitive abilities of the experimental mice. MWM was performed as previously described^37^. The water maze used was a 100-cm diameter, circular, featureless pool, filled with water containing white paint (with negligible toxicity) to a depth of 20 cm. The water was kept at room temperature (23 ± 1 °C). The pool was divided into four quadrants, and a platform was placed 1 cm below the water surface in the center of the SE quadrant. A camera recorded the tracks of the mice in the pool. Mice were placed in the water maze where they had to swim until they discovered the escape platform. The mice were allowed to briefly rest on the platform before being made to return to the water for a second attempt. This inter-trial period was ≤60 s. If a mouse was a floater (couldn’t or wouldn’t swim), the record of the mouse was deleted. The mice were trained for several days, by the end of which they had learned to swim directly to the platform. We used “escape latency” as the learning biomarker, and “swimming time in the southeast (SE) quadrant (search-to-platform area)” as the biomarker for memory. The learning biomarker was used to measure learning ability from the 6th to the 12th day, and the memory biomarker we used to evaluate memory in the “search-to-platform area” test on the 14th day.

The mice started their training exercises 4 hours after the daily exposure regime. This training included releasing each mouse from the northeast, northwest, and southwest quadrants, near to and facing the wall of the maze. Each mouse was subjected to three trials per day. Each training session lasted no more than 60 seconds. If, within 60 seconds, a mouse could not locate the platform, its escape latency was recorded as being 60 seconds, it was then led to the platform where it stayed for 30 seconds. The 7 day training period was used for the mice to learn how to find the hidden platform, and to be consequently used to evaluate mouse learning abilities. The mice were kept away from the water maze on the 13th day, giving them time to forget the location of the platform^38^. On the 14th day the platform was removed from the pool, the mice were put into the pool in the same quadrants as before, and then subjected to the probe trial test where a camera recorded their movement for 60 seconds.

### OFT

The locomotor activity of mice was assessed using an open field test. The methodological details are described by Davis *et al.*^39^. The apparatus consists of a square base (40 × 40 cm) surrounded by a 35-cm high wall, with the floor divided into 16 squares. The “border” area comprises the 12 outer squares, and the “center” area the remaining 4 squares in the center. On the 9th day, each mouse was placed individually in the center of the open-field apparatus. Testing was conducted over a 5 min (300 s) period, and mouse activity recorded using a video tracking system. After the 5 min test, the defecation number (the number of droppings) for each mouse was recorded. The walls and floors of the apparatus were cleaned thoroughly with 10% ethanol between tests.

### Brain histological assay

The mice were sacrificed by cervical dislocation 24 hours after the final intragastric intubation on day 14. The brains were removed and put in a fixative solution of saturated 2, 4, 6-trinitrophenol:formalin:glacial acetic acid [15:5:1 v/v/v] for 24 h at room temperature. H&E- and Nissl-stained slices were then prepared as described previously^40^,^41^. The stained slices were embedded in paraffin, sectioned into 10-μm slices, and examined using a DM 4000B microscope (Leica, Berlin, Germany). A non-stained region was selected and used as the background. The tissue sections were qualitatively examined in a blinded fashion, by two experienced pathologists.

### Immunohistochemical Assay

The coronal sections were cut at Bregma −3.8 mm, sections of brain tissue (3 sections were quantified per mouse) were quenched of endogenous peroxides using 3% H_2_O_2_. They were then boiled in sodium citrate (0.01 mol/L, pH 6.0) to retrieve antigens so as to unmask the antigen epitopes, following which the sections were permeabilized with 0.2% Triton X-100 for 10 min, and blocked with 10% goat serum for a further 20 min at room temperature. Sections were then incubated with diluted primary antibodies (rabbit anti-caspase-3 antibody or rabbit anti-GFAP antibody, 1:200 dilution) overnight at 4 °C. Slides were washed with PBS and incubated with a secondary antibody (goat anti-rabbit IgG; 1:200 dilutions) for 30 min at 37 °C. Caspase-3 was detected with a rabbit IgG peroxidase conjugated streptavidin-biotin complex (SABC-POD) kit, followed by incubation with a diaminobenzidine (DAB) kit. Caspase-3 and GFAP were determined by immunohistochemical staining (brown color stain). Immunostained sections were viewed under a DM 4000B microscope (Leica, Berlin, Germany) (3 images were quantified from each section). Using Image-Pro Plus 6.0 software (Media Cybernetics, Bethesda, MD, USA), GFAP or Caspase-3 staining intensity was the average optical density. A non-stained region was selected and used as the background. All tissue sections were examined blind and qualitatively assessed by two experienced pathologists.

### Tissue sample preparation

Mice brains were weighed in a fully automatic electronic balance. The tissue was put in 10 mL/g ice-cold 1× phosphate-buffer saline (PBS, pH = 7.5) and homogenized using a glass homogenizer. The homogenate was then centrifuged at 10,000 × g for 10 min at 4 °C. Supernatants were collected and stored at –70 °C until needed for determining oxidative stress levels and inflammation. The protein concentration of the supernatant was determined using the Lowry assay^42^.

### Detection of ROS

ROS levels were monitored using DCFH-DA^43^,^44^. Briefly, the supernatants were diluted 100-fold in PBS (pH = 7.5). 100 μL of this diluted solution was removed to a 96-well microplate, and 100 μL of 10 μmol/L DCFH-DA was added. The reaction mixture was kept in the dark for 30 min at 37 °C. Fluorescence intensity was then measured at an excitation wavelength of 488 nm and an emission wavelength of 525 nm by a fluorescence reader (FLx 800; BioTek Instruments, Winooski, VT, USA).

### GSH depletion assay

DTNB and glutathione (GSH) react to generate a yellow compound, 2-nitro-5-thiobenzoic acid (TNB). Once the proteins have been precipitated, the pH needs to be adjusted to 7.5 to enable the color change reaction with DTNB (60 μg/mL). Samples were analyzed using a microplate reader to measure absorbance at 412 nm^45^. Based on the standard curve, GSH concentration was determined to be (nmol/L) = OD_412_/0.0023 R^2 ^= 0.997.

### Analysis of SOD activity

SOD activity was determined with a SOD assay kit. Superoxide anions are generated by a xanthine and xanthine oxidase reaction system, and these anions can oxidize hydroxylamine to form nitrites that turn purple through the action of a chromogenic agent. SOD inhibits the superoxide anion and decreases the production of nitrites, thus reducing absorbance, the absorbance used for the spectrophotometric analysis at 550 nm wavelength.

### 8-OH-dG assay

An ELISA kit was used to measure the 8-OH-dG concentration in the brain supernatant. The kit was used according to the manufacturer’s instructions, sensitivity of the ELISA kit used was 0.5 ng/mL.

### DPC determination

A KCl-sodium dodecyl sulfate (SDS) assay was used to determine DPC levels^46^. The DPC concentrations in the mice brains were measured using a previously described procedure^47^.

### Analysis of TNF-α and IL-1β content

TNF-α and IL-1β content was measured using ELISA kits according to the manufacturer’s instructions. The sensitivities of the ELISA kits were 8 pg/mL for TNF-α and 80 pg/mL for IL-1β.

### Statistical analysis

Statistical analysis was carried out using analysis of variance (ANOVA). The graphs were generated using Origin 8.0 software (OriginLab, Berkeley, CA, USA). Statistical analyses were carried out using SPSS (SPSS, Chicago, IL, USA) software, version 13.0. A repeated-measures ANOVA followed by a Tukey test was used in the analysis of MWM escape latencies. A one-way ANOVA followed by a Tukey test was used to analyze all other data. A *P*-value of <0.05 was considered to be significant.

## Additional Information

**How to cite this article**: Ma, P. *et al.* Cognitive deficits and anxiety induced by diisononyl phthalate in mice and the neuroprotective effects of melatonin. *Sci. Rep.* **5**, 14676; doi: 10.1038/srep14676 (2015).

## Supplementary Material

Supplementary Information

## Figures and Tables

**Figure 1:**
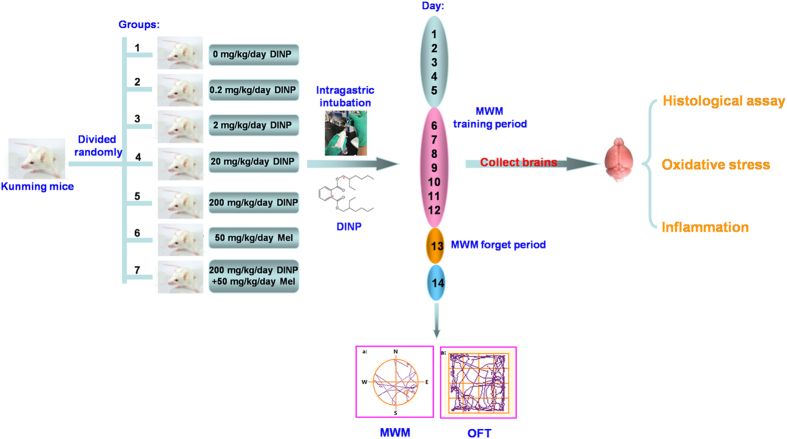
Experimental protocol. (Figure 1 was made by Xudong Liu and the different photographs of the mice were originally taken by Xudong Liu).

**Table 1:** The average optical density of the immunohistochemistry assay.

**Group**	**Caspase-3**		**GFAP**	
	**CA_1_ region**	**Cerebral cortex**	**CA_1_ region**	**Cerebral cortex**
0 mg/kg/day	0.0414 ± 0.0046	0.0446 ± 0.0060	0.0092 ± 0.00192	0.00864 ± 0.00238
0.2 mg/kg/day	0.0438 ± 0.0054	0.045 ± 0.0089	0.00842 ± 0.00498	0.0092 ± 0.00286
2 mg/kg/day	0.0438 ± 0.0083	0.0458 ± 0.0048	0.01 ± 0.00225	0.0095 ± 0.00255
20 mg/kg/day	0.138 ± 0.00462 (***P *< 0.01)**	0.2174 ± 0.02211 (***P *< 0.01)**	0.0108 ± 0.00356	0.0212 ± 0.00192 (***P *< 0.01)**
200 mg/kg/day	0.254 ± 0.00462 (***P *< 0.01)**	0.296 ± 0.03847 (***P *< 0.01)**	0.0208 ± 0.00192 (***P *< 0.01)**	0.0198 ± 0.00259 (***P *< 0.01)**

*P *< 0.01: Compared with the 0 mg/kg/day group.

**Table 2:** The average optical density of the immunohistochemistry assay.

**Group**	**Caspase-3**		**GFAP**	
	**CA_1_ region**	**Cerebral cortex**	**CA_1_ region**	**Cerebral cortex**
200 mg/kg/day	0.254 ± 0.00462	0.296 ± 0.03847	0.0208 ± 0.00192	0.0198 ± 0.00259
50 mg/kg/day Mel	0.0422 ± 0.00327	0.136 ± 0.0305	0.0146 ± 0.00365	0.012 ± 0.00255
DINP+Mel	0.134 ± 0.02966 (***P *< 0.01)**	0.086 ± 0.02408 (***P *< 0.01)**	0.018 ± 0.00158 (***P *< 0.05)**	0.009 ± 0.00224 (***P *< 0.01)**

*P *< 0.01/*P *< 0.05: Compared with the 200 mg/kg/day group.

**Table 3:** The equipment used for behavioral analysis.

**Equipment (model)**	**Manufacturer**	**Software used**	**Bio-effects tested**
Morris Water Maze	Wuhan Yi-Hong Sci&Tech. Co.,Ltd (China)	ANY-MazeTM (Stoeling Co. USA)	Cognitive ability
Open-field test	Wuhan Yi-Hong Sci&Tech. Co.,Ltd (China)	ANY-MazeTM (Stoeling Co. USA)	Anxiety emotion
