# Supplementary material for: Cognitive deficits and anxiety induced by diisononyl phthalate in mice and the neuroprotective effects of melatonin
Source: Sci Rep. 2015 Oct 1;5:14676. doi: 10.1038/srep14676 (PMC4589782; doi:10.1038/srep14676)

**Supplementary Information**

**Cognitive deficits and anxiety induced by diisononyl phthalate in mice and the neuroprotective effects of melatonin**

Ping Ma^1, #^, Xudong Liu^2, 4, #^, Jiliang Wu^1^, Biao Yan^2^, Yuchao Zhang^2, 4^, Yu Lu^2^, Yang Wu^2^, Chao Liu^1^, Junhui Guo^2^, Eewa Nanberg^3^, Carl-Gustaf Bornehag^3^, *, Xu Yang^2^, *

^1^ Hubei Province Key Laboratory on Cardiovascular, Cerebrovascular and Metabolic Disorders, Hubei University of Science and Technology, Xianning 437100, China. ^2^ Lab. of Environmental Biomedicine, Hubei Key Laboratory of Genetic Regulation and Integrative Biology, College of Life Science, Central China Normal University, Wuhan 430079, China. ^3^ Department of Health Sciences, Karlstad University, Karlstad, Sweden. ^4^ Department of Food science and Engineering, Moutai University, Renhuai 564500, China.

***Correspondence to Xu Yang (E-mail):** yangxu@mail.ccnu.edu.cn**; Carl-Gustaf**

**Bornehag (E-mail):** [carl-gustaf.bornehag@kau.se](mailto:carl-gustaf.bornehag@kau.se)**.**

**^#^These authors contributed equally to this work.**


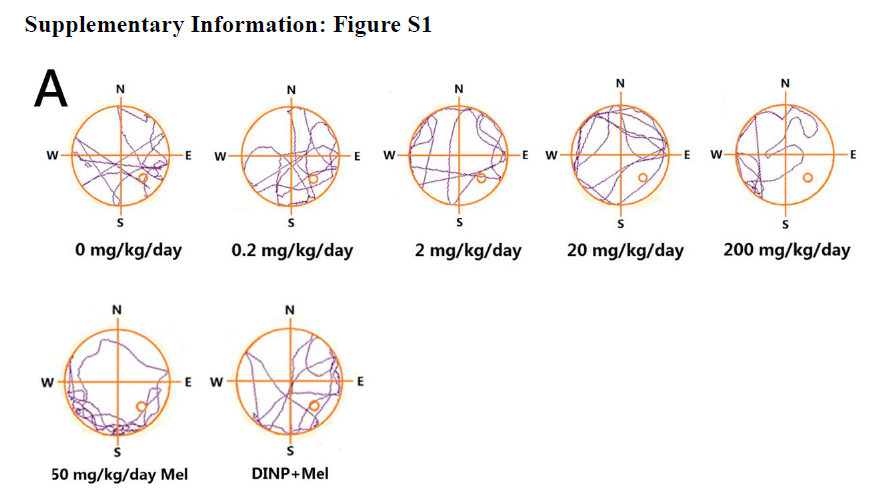


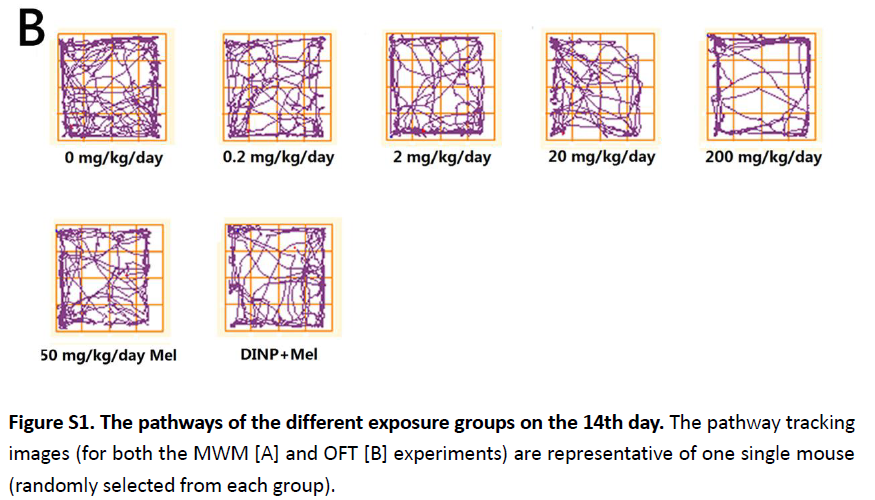

Supplement: Supplementary Information [file srep14676-s1.doc]
